# Is Risk Malignancy Index a Useful Tool for Predicting Malignant Ovarian Masses in Developing Countries?

**DOI:** 10.1155/2015/951256

**Published:** 2015-06-21

**Authors:** Aliya B. Aziz, Nida Najmi

**Affiliations:** Department of Obstetrics and Gynaecology, Aga Khan University Hospital, Karachi 74800, Pakistan

## Abstract

*Introduction*. Risk of Malignancy Index (RMI) is widely studied for prediction of malignant pelvic masses in Western population. However, little is known regarding its implication in the developing countries. The objective of this study is to determine how accurately the RMI can predict the malignant pelvic masses. *Materials and Methods*. The study is a retrospective review of patients attending the gynecological clinic between January 2004 and December 2008 with adnexal masses. Information on demographic characteristics, ultrasound findings, menopausal status, CA125, and histopathology was collected. RMI score for each patient in the study group was calculated. *Results*. The study group included a total of 283 patients. Analysis of the individual parameters of RMI revealed that ultrasound was the best predictor of malignancy with a sensitivity, specificity, and positive likelihood ratio of 78.3%, 81.5%, and 4.2, respectively. At a standard cut-off value of 250, RMI had a positive likelihood ratio of 8.1, while it was 6.8 at a cut-off of 200, albeit with comparable sensitivity and specificity. *Conclusion*. RMI is a sensitive tool in predicting malignant adnexal masses. A cut-off of 200 may be suitable in developing countries for triaging and early referral to tertiary care centers.

## 1. Introduction

Ovarian masses are a frequent cause of gynecological consults and are often detected during imaging studies or exploratory surgery for evaluation of abdominal or pelvic pain syndromes. They occur across age groups and could result from benign or malignant disease. With more than 250,000 new cases reported every year, ovarian malignancies represent the fourth commonest cause of cancer deaths worldwide [1]. They also have the lowest 5-year survival rate (30–50%) among all gynecological cancers [2]. A recent report indicated an increasing incidence of ovarian cancers in the developing world, compared to the developed countries [3].

Early identification of ovarian carcinomas and referral to a gyneco-oncologist can facilitate accurate staging of the disease and optimal cytoreductive treatment, enhancing patient survival [4, 5]. Histopathology remains the diagnostic gold standard for this cancer, and a definitive biomarker has not been identified yet. Risk of Malignancy Index (RMI), which considers the serum CA125 level, menopausal status, and ultrasonographic findings in predicting malignant pelvic masses, is widely employed in the developed countries [6]. However, its utility in risk prediction in the developing countries is currently unknown.

The present study evaluated how accurately the RMI can predict the risk of malignant pelvic masses, among patients with an ovarian mass.

## 2. Material and Methods

After the approval of our institutional review board we conducted a retrospective review of the case files of patients with adnexal masses who attended the Gynecological Clinic at the Aga Khan University, Karachi, Pakistan, between January 2004 and December 2008. The International Classification of Diseases, 9th Revision, Clinical Modification (ICD-9-CM) criteria were used to identify adnexal masses. Patients with advanced disease were excluded from the study. We collected information on demographic characteristics, ultrasonographic findings, menopausal status, serum CA125 level, and histopathology. The RMI for each patient was calculated using the standard formula [6].

## 3. Results

### 3.1. Demographic Data

The study group consisted of a total of 283 patients. The age of the patients varied from 8 to 85 years (mean, 38.6 years). Premenopausal patients predominated in our study with 227 (80.8%) cases, while 54 (19.2%) of the affected patients were in the postmenopausal group.

### 3.2. Ultrasonography Findings

Two hundred and seven (73.7%) patients had a transabdominal ultrasonography for diagnosis, while transvaginal ultrasonography detected the disease in 74 (26.3%) cases.  Table 1 shows the summary of ultrasound findings in our patients.

#### 3.2.1. Laterality of Lesion

The investigation revealed a unilateral cyst in 252 (89.7%) cases, while 29 (10.3%) had bilateral cysts.

#### 3.2.2. Loculation

The lesions were multilocular in 166 (59%) patients and unilocular in 115 (41%).

#### 3.2.3. Echogenicity

Solid areas were absent in the lesions in 196 (69.8%) patients, while these were detected in 85 (30.2%) patients.

#### 3.2.4. Evidence of Metastasis

The majority of patients (272, 96.8%) had no evidence of metastasis, while 9 (3.2%) had metastatic disease.

#### 3.2.5. Presence of Ascites

Ascites was present in only 19 (6.8%) patients.

#### 3.2.6. Ultrasound Score

We assigned scores of 0 (absence of specific findings), 1 (presence of one finding), or 3 (two or more findings) to the subjects, depending on the ultrasound findings. One hundred and nineteen (42.3%) cases had an ultrasound score of 1, while lesions of 88 (31.3%) and 74 (26.3%) patients were scored 0 and 3, respectively. Of the 207 (73.6%) patients with an ultrasound score 1, 196 (69.7%) had benign disease, while 8 (2.8%) and 3 (1%) had malignant and borderline disease, respectively. Seventy-four (26.3%) patients in our series had an ultrasound score of 3, and among them, 41 (14.5%) had benign, 29 (10.3%) had malignant, and 4 (1.4%) had borderline disease, respectively.

#### 3.2.7. Ovarian Size

Ovarian size varied from 3 to 73 cm (mean, 10.5 cm).

### 3.3. Histopathology Findings

As shown in Table 2, 237 (84.3%) patients had benign lesions, while 37 (13.2%) had a malignant disease. Seven (2.5%) patients under 60 years of age had borderline lesions. One hundred and thirty-nine (49.4%) of the benign tumours occurred in patients aged 20 to 39 years, and 60 (21.3%) cases were in those aged 41–59 years. Patients aged ≤20 years and ≥60 years reported 24 (8.5%) and 14 (4.9%) cases of benign disease, respectively. Malignant disease peaked in the age group 40–59 years with 21 (7.4%) cases, while 10 (3.5%), 4 (1.4%), and 2 (0.7%) cases occurred among patients aged ≥60 years, 20–39 years, and ≤20 years, respectively. Three cases of borderline disease occurred in the age groups 21–39 and 40–59 years, and one (0.3%) case was in a woman aged ≤20 years, while such lesions were not detected in women ≥60 years. Two hundred and three (72.2%) of the 227 premenopausal patients had benign disease, while 18 (6.4%) had malignant, and 6 (2.1%) had borderline lesions. Among the 54 (19.2%) postmenopausal patients, 34 (12%) had benign disease, while 19 (6.7%) and 1 (0.3%) had malignant and borderline disease, respectively.

### 3.4. Serum CA125 Levels

The serum CA125 levels in the patients varied from 1.2 to 6803 U/mL (mean, 197 U/mL) (Table 2). One hundred and seventy-six (62.6%) patients had a serum CA125 level less than 35 U/mL, while the levels were higher in 105 (37.3%) patients. Among the patients with CA125 levels greater than 35 U/mL, 75 (26.6%) had benign disease, 26 (9.2%) had malignant, and 4 (1.4%) had borderline lesions. One hundred and sixty-two (57.6%) patients with CA125 levels less than 35 U/mL had benign lesions, while 11 (3.9%) had malignant, and 3 (1%) had borderline disease.

### 3.5. RMI

The RMI was calculated according to a standard formula (Jacobs et al., 1990). The RMI scores of the patients varied from 1.9 to 32364 (mean, 601.1 ± 3196.3) (Table 2). Two hundred and forty-five (87.1%) patients had an RMI score less than 250, while 36 (12.8%) had scores above 250. Twenty of the patients with RMI scores ≥250 had malignant disease, while 14 had benign and 2 had borderline lesions. Among patients with RMI scores less than 250, 223 (79.3%) had benign disease, while 17 (6%) and 5 (1.7%) had malignant and borderline lesions, respectively.

### 3.6. Risk Stratification Based on RMI Scores

We assessed the distribution of benign, borderline, and malignant ovarian cancers when the patients were categorized based on their RMI scores.

In order to identify the RMI score that was an effective risk predictor, we studied the sensitivity and specificity of RMI scores at four levels, namely, ≤100, ≤150, ≤200, and ≤250. The sensitivity, specificity, and positive and negative predictive values of RMI score at each of these levels are summarized in Table 3.

One hundred and twenty (42.7%) patients had RMI scores ≤25, among whom 117 (41.6%) had benign disease, 3 (1%) had malignant disease, and none had borderline lesions. The scores ranged from 25.1 to 249 in 125 (42.7%) patients. In this group, 106 (37.7%) patients had benign disease and 5 (1.7%) had borderline disease, while 14 (4.9%) had malignant disease. In the third group with RMI scores ≥ 250, 20 (7.1%) had malignant disease, 2 (0.7%) had borderline disease, and 14 (4.9%) had benign disease.

To find out the RMI score that could most effectively classify the disease, we calculated the sensitivity, specificity, positive predictive value, negative predictive value, and the likelihood ratios at RMI cut-off levels of 100, 150, 200, and 250. A comparison of the diagnostic indices with these cut-offs is shown in Table 3.

As shown in Table 3, an RMI of 250 yielded the ideal combination of sensitivity (54.05), specificity (93.4), positive predictive value (55.5), negative predictive value (93.06), and positive (8.1) and negative (0.49) likelihood ratios. Though cut-offs of 100 and 150 showed higher sensitivity in detecting malignant disease, they had lower specificity, positive predictive value, and likelihood ratios, compared to 250.

We also compared the diagnostic performance of RMI scores >250 against CA125 levels >35 U/mL, ultrasound score of 3 and menopausal score of 3.  Table 4 summarizes the findings from this analysis. Among the three criteria, an ultrasound score of 3 had the highest sensitivity (78.3%), while an RMI score ≥250 had the highest specificity (93.4%). The latter also had the highest positive predictive value of 55.5%, while negative predictive value was highest for an ultrasound score of 3 (96.1%). The positive likelihood ratio was highest for RMI score ≥250, while a score of 100 had the least negative likelihood ratio (0.39).

## 4. Discussion

About 10% of women undergo exploratory surgery for evaluation of ovarian masses during their lifetime [7]. Prompt identification of ovarian malignancies and referral to a gyneco-oncologist can enhance the patient survival rates [8], but a single method which can accurately predict ovarian malignancy is still unavailable. Herein we report that the multiparametric RMI score can be a useful tool in prediction of malignant ovarian disease, in low-resource settings.

The mean age of the patients with ovarian mass in our study was 36.87 years (range, 8 to 85 years). This is slightly higher than that reported in a similar study by Akdeniz et al. in 2009 [9].

In our study, 13.2% of the patients with an ovarian mass had malignant disease. Thirty-five percent of malignancies occurred in postmenopausal patients and 7.9% among the premenopausal patients. The data seem to agree with earlier reports of similar incidence rates and preponderance in postmenopausal patients [9–12].

Ultrasonography is widely appreciated as the best imaging method for evaluation of ovarian pathology. Several groups have reported higher sensitivity, specificity, and positive predictive values for this method (Agarwal et al., 2011, and references therein). In our study, an ultrasound score of 3 had the highest sensitivity (78.3%) and negative predictive value (96.1%) and the least negative likelihood ratio (0.26), among the parameters evaluated.

Several candidate biomarkers and their combinations have been employed in assessing the risk of ovarian malignancies, albeit with varying efficiency [13]. Serum CA125 level is widely appreciated as a useful biomarker for estimating the risk of ovarian cancer, though other gynecological pathology can also increase its levels. Myers et al. [14] have earlier reported sensitivity and specificity of less than 80%, for this marker, in the prediction of ovarian cancers. Simsek et al. (2014) [15] reported a sensitivity of 78.6% and specificity of 63.5% for a CA125 cut-off of 35 U/mL. Another report indicated a sensitivity of 88% and specificity of 97% for CA125 at a higher cut-off of 88 U/mL [12]. In our study, CA125 levels ≥35 U/mL had a sensitivity of 70.2%, specificity of 67.6%, positive predictive value of 24.7%, negative predictive value of 93.7, and positive and negative likelihood ratios of 2.1 and 0.44, respectively. We suggest that a higher prevalence of inflammatory and nonspecific uterine and ovarian pathology might have contributed to elevated CA125 levels in the majority of our patients and thus its low diagnostic performance in the detection of malignant ovarian disease.

Rao (2014) [16] has recently reported higher sensitivity, specificity, and positive and negative predictive values for a postmenopausal score of 3. In our study, this parameter had a higher specificity and negative predictive value, but lower sensitivity and positive predictive values in assessing malignancy risk.

RMI was first proposed by Jacobs et al. and is calculated from the serum CA125 antigen level, menopausal status, and ultrasonographic findings [6]. Several retrospective and prospective studies have reported it to be the best available tool for triage and referral of ovarian malignancies [17, 18]. Its utility as a diagnostic tool depends on the prevalence of malignancy in the study population [15]. We observed a low prevalence of malignancy (13.2%) among our study group, significantly lesser than some of the earlier reports of 30–43% [6, 17, 19].

Jacobs et al. (1990) [6], studying 143 patients, reported a sensitivity of 85.4% and specificity of 96.9% for this method, with a cut-off of 200. Subsequently, several groups have reported its superior sensitivity and specificity in estimating the risk of ovarian malignancy, compared to other parameters [19–25]. The RMI cut-offs in many studies ranged from 25 to 250 (reviewed in Geomini et al., 2009) [18]. Most studies reported an increased diagnostic accuracy and performance with an RMI cut-off of 200 [6, 16, 19, 20, 22, 24, 26–32]. A recent study reported a sensitivity of 89.5%, specificity of 96.2%, positive predictive value of 77.3%, and negative predictive value of 98.4% [11], when a higher RMI cut-off of 238 was used for the screening. Yamamoto et al. (2009) [25] reported a sensitivity and specificity of 75% and 91%, respectively, using a cut-off of 450. The best performance in the present study was seen with an RMI cut-off of 250, and the low sensitivity (54.5%) and high specificity (93.4%) observed were comparable to the majority of earlier reports that employed a similar cut-off [6, 19, 20, 22, 26, 29–35].

We conclude that, in the absence of a definitive biomarker, the multiparametric Risk of Malignancy Index serves as a very useful tool for identification of malignant ovarian disease and their prompt triage and referral to expert care.

## Figures and Tables

**Table 1:** Summary of ultrasound findings in the study.

	Frequency	Percentage (%)
Transabdominal scan	207	73.7
Transvaginal scan	74	26.3

Unilateral cyst	252	89.7
Bilateral cyst	29	10.3

Unilocular cyst	115	41.0
Multilocular cyst	166	59.0

Presence of solid areas	85	30.2
Absence of solid areas	196	69.8

Evidence of metastasis present	9	3.2
Evidence of metastasis absent	272	96.8

Presence of ascites	19	6.8
Absence of ascites	262	93.2

Ultrasound score 0	88	31.3
Ultrasound score 1	119	42.3
Ultrasound score 3	74	74

**Table 2:** Distribution of cases in the study.

	Benign (%)	Borderline (%)	Malignant (%)	Total (%)
Histopathology	237 (84.3)	7 (2.5)	37 (13.2)	281

Age (years)				
≤20	24 (8.5)	1 (0.3)	2 (0.7)	27 (9.6)
20–39	139 (49.4)	3 (1.0)	4 (1.4)	146 (51.9)
40–59	60 (21.3)	3 (1.0)	21 (7.4)	84 (29.8)
≥60	14 (4.9)	0 (0)	10 (3.5)	24 (8.5)

Premenopausal	203 (72.2)	6 (2.1)	18 (6.4)	227 (80.7)
Postmenopausal	34 (12.0)	1 (0.3)	19 (6.7)	54 (19.2)

Ultrasound score 1	196 (69.7)	3 (1.0)	8 (2.8)	207 (73.6)
Ultrasound score 3	41 (14.5)	4 (1.4)	29 (10.3)	74 (26.3)

CA125 ≥ 35	75 (26.6)	4 (1.4)	26 (9.2)	105 (37.3)
Ca125 < 35	162 (57.6)	3 (1.0)	11 (3.9)	176 (62.6)

RMI groups				
≤25	117 (41.6)	0 (0)	3 (1.0)	120 (42.7)
25.1–249.9	106 (37.7)	5 (1.7)	14 (4.9)	125 (44.4)
≥250	14 (4.9)	2 (0.7)	20 (7.1)	36 (12.8)

**Table 3:** Diagnostic performance of the different RMI cut-offs employed.

RMI cut-off value	Sensitivity	Specificity	PPV	NPV	+LR ratio	−LR ratio
250	54.05 (20/37)	93.4 (228/244)	55.5 (20/36)	93.06 (228/245)	8.1	0.49
200	53.8 (21/39)	92.2 (225/244)	52.5 (21/40)	92.5 (225/243)	6.8	0.50
150	61.5 (24/39)	89.3 (218/244)	48.0 (24/50)	93.5 (218/233)	5.7	0.43
100	66.6 (26/39)	84.0 (205/244)	40.0 (26/65)	94.0 (205/218)	4.1	0.39

**Table 4:** Diagnostic performance of the criteria evaluated.

	Sensitivity %	Specificity %	PPV (%)	NPV (%)	+ve likelihood ratio	−ve likelihood ratio
RMI ≥ 250	54.05 (20/37)	93.4 (228/244)	55.5 (20/36)	93.06 (228/245)	8.1	0.49
CA125 ≥ 35	70.2 (26/37)	67.6 (165/244)	24.7 (26/105)	93.7 (165/176)	2.1	0.44
Ultrasound score 3	78.3 (29/37)	81.5 (199/244)	39.1 (29/74)	96.1 (199/207)	4.2	0.26
Menopause score 3	51.3 (19/37)	85.6 (209/244)	35.1 (19/54)	92.0 (209/227)	3.5	0.56
